# Antidiarrheal and Antioxidant Activities of Methanol Extract of* Bryophyllum pinnatum* Leaf Harvested from South-Eastern Nigeria in Mice

**DOI:** 10.1155/2018/6810620

**Published:** 2018-05-29

**Authors:** Samuel O. Onoja, Ginika Q. Ihejirika, Oluchi N. Nwankudu, Yusuf N. Omeh, Maxwell I. Ezeja

**Affiliations:** ^1^Department of Veterinary Physiology and Pharmacology, College of Veterinary Medicine, Michael Okpara University of Agriculture, PMB 7267, Umudike, Abia State, Nigeria; ^2^Department of Biochemistry, College of Natural Sciences, Michael Okpara University of Agriculture, PMB 7267, Umudike, Abia State, Nigeria

## Abstract

*Bryophyllum pinnatum* belongs to the family Crassulaceae and it is commonly used in the ethnomedical practices. This study investigated the antidiarrheal and antioxidant properties of methanol extract of* Bryophyllum pinnatum *leaf harvested from South-Eastern Nigeria in mice. Cold maceration method in 80% methanol was adopted in the extract preparation. The 2,2-diphenyl-1-picrylhydrazyl (DPPH) and ferric reducing antioxidant power (FRAP) assays were used to evaluate the antioxidant property while castor oil-induced diarrhea, small intestinal transit, and enteropooling models were used for the antidiarrheal investigation. The effects of the extract (50, 100, and 200 mg/kg) were compared to distilled water (10 ml/kg) and loperamide (5 mg/kg). The extract produced concentration dependent increase in antioxidant effect in both DPPH and FRAP assay. The extract caused a significant (*p* < 0.05) reduction in mean stool output, percentage of wet stools, small intestinal transit, and intestinal fluid accumulation in the treated mice when compared to the distilled water treated mice. The study validates the use of* Bryophyllum pinnatum* in the ethnomedical management of diarrhea.

## 1. Introduction

Diarrhea is a common cause of hospitalization among children and even elderly persons in most developing countries and accounts for about 5 million deaths annually [1]. It is characterized by increased frequency, fluidity, and bowel movement of three or more times per day as well as inflammatory response and oxidative stress [2, 3]. It occurs due imbalanced in the secretory and absorptive physiologies of water and electrolyte in the gut which might be caused by microorganism (bacteria, virus, protozoa, and fungi), helminths, toxins, diet, and allergy [4]. Diarrhea is often treated with antispasmodic, antisecretory, and inhibitors of prostaglandin secretions such as loperamide, diaretyl, and atropine [3, 5]. These drug are associated with side effects such constipation, drowsiness, and colorectal cancer which limit their usage [5]. In the tropics several natural occurring compounds are employed in the treatment of diarrhea and other gastrointestinal disorders [1]. In South-Eastern Nigeria,* Bryophyllum pinnatum *is among the medicinal plants included in herbal preparations meant for diarrhea control.

*Bryophyllum pinnatum* belongs to the family Crassulaceae and the common names include life plant, love plant, miracle leaf, and Canterbury bells. It is widely distributed in tropical Africa, America, Hawaii, India, China, Australia, and Madagascar [6]. The leaf is extensively used in folk medicine as an astringent, emollient, hemostatic, carminative, disinfectant, and tonic. It is also used in conditions such as hematemesis, hemorrhoids, menstrual pain, wounds, boils, sloughing ulcers, ophthalmia, burns, scalds, diarrhea, and dysentery [7]. Some biological active compounds, including alkaloids, triterpenes, lipids, flavonoids, glycosides, kaempferol rhamnoside, bufadienolides, phenols, and organic acids have been isolated from the plant [6–8]. The leaves of* B. pinnatum* have been reported to possess hepatoprotective and antineoplastic [6, 9], anti-asthmatic and antitussives [9, 10], antidiabetic [10], antihypertensive [11], antimicrobial [12], anti-inflammatory and analgesic [13, 14], and antiulcer [15] activities. The antidiarrheal activity of* B. Pinnatum *harvested from Southwest ecological zone has been reported [16]. The ecological factors such as rainfall, soil type, light intensity, and humidity affect the phytochemical composition of medicinal plants as well as the pharmacological activities [17]. There is paucity of data on the antidiarrheal effects of* B. pinnatum* leaf grown in South-Eastern Nigeria ecological zone. The study investigated the antidiarrheal effects of methanol extract of* B. Pinnatum *leaf harvested from South-Eastern Nigeria in mice.

## 2. Materials and Method

### 2.1. Plant Collection and Identification

Fresh leaves of* Bryophyllum pinnatum* were collected from Ugba Junction in Isiala Ngwa South Local Government Area of Abia State, South-Eastern Nigeria in June 2016 (rainy season) and were confirmed* Bryophyllum pinnatum *by a plant taxonomist, Dr. M. C. Dike of College of Natural Resources and Environmental Management, Michael Okpara University of Agriculture, Umudike. A voucher specimen with catalogue number MOUAU/CVM/VPP/2016/44 was deposited in the Department of Veterinary Physiology and Pharmacology herbarium for reference.

### 2.2. Chemicals and Drugs

Ascorbic acid (Hopkin and Williams, England), loperamide hydrochloride (Xian-Janssen Pharmaceutical Ltd., China), charcoal, castor oil, gum acacia (Merck, Germany), 2,2-diphenyl-2-picrylhydrazyl (DPPH), methanol, sodium acetate trihydrate, glacial acetic acid, 2,4,6-tripyridyl-s-triazine (TPTZ), and hydrochloric acid purchased from Sigma-Aldrich, Germany were used for the study.

### 2.3. Extraction of Plant Material

The leaves of* Bryophyllum pinnatum* were dried under room temperature on a laboratory bench and were ground into a coarse powder using manual grinder (Corona, China). The powdered material was weighed using an electronic balance (PI 303 model, China). The plant material (150 g) was macerated with 80% methanol for 48 hours with intermittent shaking every 3 hours. The resulting mixture was filtered with Whatman No. 1 filter papers and was dried in a hot air oven at 40°C. The extract was stored in a refrigerator at 4°C until used. The percentage yield (w/w) of the* Bryophyllum pinnatum* extracts (BPE) was calculated using the formula below:

(1) Percentage  yield  w/w=weight  of  extract÷weight  of  starting  plant  materialweight  of  starting  plant  material×1001

### 2.4. Animals

Ninety (90) mice (28–34 g), aged 8–10 weeks, sourced from the laboratory animal unit of the Department of Veterinary Physiology and Pharmacology, Michael Okpara University of Agriculture Umudike, Abia State, were used for the study. The animals were housed in aluminum cages at room temperature and under natural light/darkness cycles. The mice were supplied with clean drinking water and fed with standard commercial pelleted grower feed (Vital feed® Nigeria)* ad libitum*. The mice were acclimatized for two weeks prior to the study. They were maintained in accordance with the recommendations of the Guide for the care and use of laboratory animals [18] and the experimental protocol was approved by the institution's ethical committee.

### 2.5. Antidiarrheal Study

#### 2.5.1. Castor Oil-Induced Diarrhea

We followed the methods of Onoja and Udeh [19]. Briefly, thirty (30) mice were divided into 5 groups (A–E) of 6 mice per group. They were fasted for about 10 h with free access to water, but water was removed 2 h prior to the test. Group A received 10 mL/kg of distilled water and Group B received loperamide (5 mg/kg) while Groups C, D, and E received 50, 100, and 200 mg/kg of BPE, respectively. One hour after administration of drugs, diarrhea was induced by administering 0.3 ml of castor oil orally. The animals were placed individually in cages lined with white paper. The numbers of both wet and dry stool droppings were counted at 1 h interval over a period of 4 h. The mean percentage of wet stool passed by the treated groups was compared with that of the control group [20].

(2) %  wet  stools=total  number  of  wet  stooltotal  number  of  both  wet  and  dry  stool×1001

#### 2.5.2. Small Intestinal Transit

The modified method of Chime [21] as described in Onoja and Udeh [19] was followed in this experiment. Briefly, thirty mice were randomly assigned to five groups (A–E) of six mice each and were fasted for 10 h before the experiment. Group A received 10 mL/kg of distilled water and Group B received loperamide (5 mg/kg) while Groups C, D, and E received 50, 100, and 200 mg/kg of BPE, respectively. The standard charcoal meal (5% of activated charcoal suspended in 5% of gum acacia) was administered to all the animals 1 h after treatment. The animals were sacrificed 30 min after administration of charcoal meal by cervical dislocation and the intestines were immediately isolated and ligated at the pyloric sphincter and ileocecal junction. The small intestinal transit was expressed as percentage of distance travelled by the charcoal meal relative to the total length of the small intestine from the pyloric sphincter to the ileocecal junction.

(3) %  distance  travelled=distance  travelled  by  the  charcoal  mealFull  length  of  the  small  intestine×1001%  inhibition=%  distance  travelled  of  control  group−%  distance  travelled  of  treated  group%  distance  travelled  of  control  group×1001

#### 2.5.3. Enteropooling

The method of Hassan et al. [22] as described in Onoja and Udeh. [19] was followed in this study. Briefly, thirty mice were randomly divided into five groups of six mice each and were fasted for 10 h before the experiment. Group A received 10 mL/kg of distilled water and Group B received loperamide (5 mg/kg) while Groups C, D, and E received 50, 100, and 200 mg/kg of BPE, respectively. One h after the treatment, the animals were sacrificed by cervical dislocation and laparotomized and the intestines were immediately isolated and ligated at the pyloric sphincter and ileocecal junction. The small intestines were weighed, the content of each intestine was milked out, and the empty intestines were reweighed. The difference in weight between the full and empty intestines was recorded as the weight of the intestinal content.

(4) Weight  of  intestinal  content=wt  of  intestine  with  content−wt  of  empty  intestine%  inhibition=wt  of  intestinal  content  of  control−wt  of  intestinal  content  of  treated  groupwt  of  intestinal  content  of  control×1001,

where wt = weight.

### 2.6. Antioxidant Study

#### 2.6.1. 2,2-diphenyl-1-picrylhydrazyl (DPPH) Photometric Assay

The DPPH radical scavenging activity of the extract was analyzed as reported by Onoja et al., [23] using spectrophotometer. The extract at various concentrations (25, 50, 100, 200, and 400 *μ*g/ml) was assayed in triplicate and ascorbic acid was also assayed as a reference standard.

#### 2.6.2. Ferric Reducing Antioxidant Power

The ferric reducing antioxidant power of BPE was evaluated as described by Onoja et al. [24]. The concentrations of 25, 50, 100, 200, and 400 *μ*g/ml of BPE in triplicate were used in the study. It was compared with ascorbic acid at 125 *μ*g/ml concentration.

### 2.7. Data Analysis

The obtained data were statistically evaluated using one-way ANOVA, followed by least significant difference test with SPSS software. The mean values were considered significant at *p* < 0.05.

## 3. Results

### 3.1. Effect of BPE and Loperamide on Castor Oil-Induced Diarrhea on Mice

The extract caused a significant (*p* < 0.05) dose-dependent decrease in the percentage of wet stools (Table 1) in the treated groups throughout the period of observation when compared with distilled water treated groups. At 4 h after diarrhea induction, the percentages of wet stool of the distilled water, loperamide, and BPE 50, 100, and 200 mg/kg were 84.62, 90.00, 83.61, 72.14, and 62.50%, respectively. The optimum antidiarrheal effect of BPE was produced at 200 mg/kg dose.

### 3.2. Effect of BPE and Loperamide on Small Intestinal Transit in Mice

The loperamide and BPE (50, 100, and 200 mg/kg) significantly (*p* < 0.05) reduced the small intestinal transit of charcoal meal in the treated mice (Table 2) when compared with distilled water treated mice. The extract did not produce dose-dependent effect. The maximal effect of the extract was observed at 50 mg/kg.

### 3.3. Effect of BPE and Loperamide on Enteropooling in Mice

The loperamide and BPE (50, 100, and 200 mg/kg) caused a significant (*p* < 0.05) reduction in the weight of the intestinal content in the treated mice when compared to the distilled water treated mice (Table 3). The BPE did not produce a dose-dependent effect. The weights of the intestinal content of the distilled water, loperamide, and BPE 50, 100, and 200 mg/kg were 0.91, 0.45, 0.60, 0.67, and 0.66 g, respectively.

### 3.4. DPPH Radical Scavenging Activities of BPE

**Figure 1:**
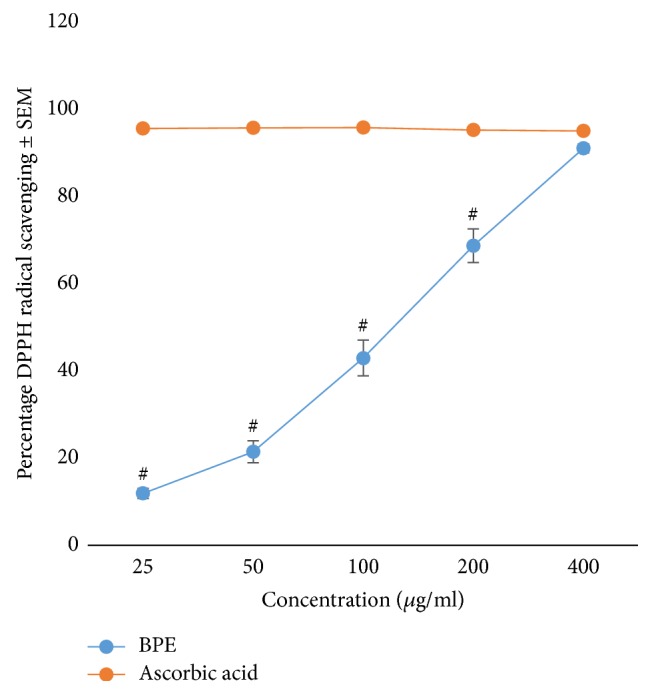
DPPH radical scavenging activities of BPE. ^#^*p* < 0.05 when compared with ascorbic acid, BPE =* Bryophyllum pinnatum* extract.

The extract produced a concentration dependent increase in percentage antioxidant activity. The optimum antioxidant activity of BPE was produced at 400 *μ*g/ml concentration (Figure 1).

### 3.5. Ferric Reducing Antioxidant Power of BPE

**Figure 2:**
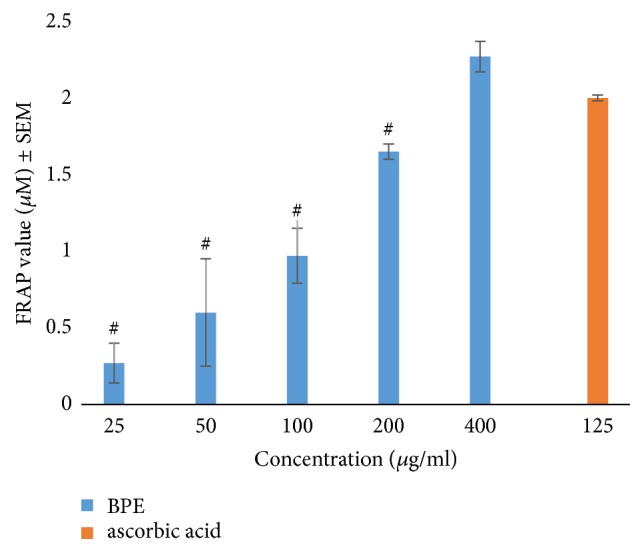
Ferric reducing antioxidant power (FRAP) of BPE. ^#^*p* < 0.05 when compared with ascorbic acid, BPE =* Bryophyllum pinnatum* extract.

The extract produced a concentration dependent increase in antioxidant power. At 400 *μ*g/ml the assay gave 2.27 (*μ*M), showing that BPE has a high antioxidant power (Figure 2).

## 4. Discussion

The antidiarrheal property of methanol extract of* Bryophyllum pinnatum *was evaluated using castor oil-induced diarrhea, intestinal transit, and enteropooling models in mice. The extract significantly (*p* < 0.05) inhibited castor oil-induced diarrhea, reduced intestinal transit, reduced intestinal fluid accumulation, and elicited potent antioxidant activities. These pharmacological activities could be mediated by the phytochemical constituents of* Bryophyllum pinnatum *[25]. The presence of phytoconstituents like alkaloids, terpenes, glycosides, and flavonoids has been reported on* B. pinnatum* and the antidiarrheal and antioxidant activities of these phytoconstituents have been reported as well [26].

The inhibition of castor oil-induced diarrhea might be linked to protection against gastric irritation and inflammation as well as reduction in prostaglandin release [27, 28]. Ricinoleic acid, the active metabolite of castor oil, causes irritation and inflammation of the intestinal mucosa which leads to increased prostaglandin release, enhanced peristalsis, and reduced reabsorption of sodium ion, chloride ion, and water from the gut which give rise to diarrhea [29]. The inhibition of prostaglandin synthesis has been incriminated in the anti-inflammatory and analgesic activities of* B. pinnatum* [13, 14].

The reduction in intestinal transit may be due to reduced peristalsis which can be attributed to relaxation of the intestinal smooth muscle [30, 31]. Drugs that relax the intestinal smooth muscles are often used as antidiarrheal agent because they inhibit intestinal hypermotility (increase peristalsis) which usually accompanies diarrhea [26]. Antidiarrheal activity of loperamide is linked to inhibition of peristalsis [32, 33]. The reduction in intestinal fluid accumulation is linked to antisecretory activity. Tannins and flavonoids present in the plant extract are reported to inhibit release of prostaglandins, thereby inhibiting motility and secretion induced by castor oil. The antidiarrheal activity of the extract may also be due to denature of proteins by tannates that make intestinal mucosa more resistant and reduce secretion [25]. The reported antimicrobial activities of* B. pinnatum* suggest that the extract could be effective in the management of susceptible microbial induced diarrhea [34, 35]. The findings of this study corroborate the antidiarrheal activities of* Combretum dolichopetalum *[19] as well as the report of Adeyemi et al. [16] on the antidiarrheal effects of* B. pinnatum* harvested from South-Western Nigerian.

The antidiarrheal effects of* B. Pinnatum *may also be associated with its potent antioxidant potential as observed in this study. Umukoro and Ashorobi reported that ascorbic acid and *α*-tocopherol reduce prostaglandin level through the inhibition of perioxidation of phospholipids and thus ameliorate castor oil-induced diarrhea [27]. The antidiarrheal and antioxidant activities of* Bryophyllum pinnatum *could be mediated by the phytochemical composition. Phytochemical investigations have shown the presence of alkaloids, triterpenes, lipids, flavonoids, glycosides, kaempferol rhamnoside, bufadienolides, phenols, and organic acids [6–8].

## 5. Conclusion

The findings of this study demonstrated the pharmacological basis for ethnomedical use of* Bryophyllum pinnatum *in diarrhea treatment. However further studies are desired toward the isolation and characterization of the active compound.

## Figures and Tables

**Table 1:** Effect of BPE and loperamide on castor oil induced diarrhea on mice.

Percentage (%) wet stool				
Treatment	1 h	2 h	3 h	4 h
Distilled water 10 ml/kg	76.66 ± 2.35	81.38 ± 2.00	82.57 ± 2.27	84.62 ± 2.23
Loperamide 5 mg/kg	0.00 ± 0.00^*∗*^	40.00 ± 16.32^*∗*^	88.75 ± 3.39	90.00 ± 2.98
BPE 50 mg/kg	48.33 ± 9.06^*∗*^	63.54 ± 11.01	83.06 ± 2.01	83.61 ± 2.18
BPE 100 mg/kg	28.33 ± 9.77^*∗*^	50.00 ± 11.57	61.66 ± 8.48^*∗*^	72.14 ± 5.13^*∗*^
BPE 200 mg/kg	20.83 ± 10.75^*∗*^	61.11 ± 2.02	63.49 ± 2.89^*∗*^	62.50 ± 4.56^*∗*^

^*∗*^ *p* < 0.05 when compared with distilled water treated group. BPE = *Bryophyllum pinnatum* Extract.

**Table 2:** Effects of BPE and loperamide on small intestinal transit in mice.

Treatment	% Distance travelled	% Inhibition
Distilled water 10 ml/kg	60.65 ± 0.46	0
Loperamide 5 mg/kg	45.10 ± 2.20^*∗*^	25.63
BPE 50 mg/kg	43.78 ± 2.96^*∗*^	27.81
BPE 100 mg/kg	58.93 ± 2.76	2.83
BPE 200 mg/kg	54.60 ± 0.49	9.97

^*∗*^ *p* < 0.05 when compared with distilled water treated group. BPE = *Bryophyllum pinnatum* extract.

**Table 3:** Effects of BPE and loperamide on the enteropooling in mice.

Treatment	WT of intestinal content (g)	% inhibition
Distilled water 10 ml/kg	0.91 ± 0.01	-
Loperamide 5 mg/kg	0.45 ± 0.07^*∗*^	50.55
BPE 50 mg/kg	0.60 ± 0.01^*∗*^	34.07
BPE 100 mg/kg	0.67 ± 0.01^*∗*^	26.37
BPE 200 mg/kg	0.66 ± 0.01^*∗*^	27.47

^*∗*^ *p* < 0.05 when compared with distilled water treated group. BPE = *Bryophyllum pinnatum* extract, WT = weight.
